# Predictive value of the dynamic systemic immune-inflammation index in the prognosis of patients with intracerebral hemorrhage: a 10-year retrospective analysis

**DOI:** 10.3389/fneur.2024.1444744

**Published:** 2024-10-08

**Authors:** Wenchao Ma, Xiaoming Wu, Lang Yang, Yumin Yang, Hao Zhang, Yan Wang, Haiying Xue, Xiaodong Long

**Affiliations:** Department of Neurosurgery, People’s Hospital of Deyang City, Deyang, China

**Keywords:** systemic immune-inflammation index, intracerebral hemorrhage, predictive value, dynamic, prognosis

## Abstract

**Background and purpose:**

The systemic immune-inflammation index (SII) is a novel immune inflammatory marker which has been proven to have excellent predictive value for many diseases. The aim of this study was to investigate the predictive value of SII at different time points after admission for functional outcome at discharge in patients with intracerebral hemorrhage (ICH).

**Methods:**

The clinical data of patients with ICH who were treated at a medical center for neurological diseases in China between October 2012 and April 2022 were analyzed in this retrospective study. The SII was calculated based on neutrophil×platelet/lymphocyte counts and collected within the first 14 days after admission to analyze the dynamic changes. Adverse outcome was defined as a modified Rankin Scale (mRS) score of 4–6 at discharge. The correlation between the SII and the outcome was assessed using univariate and multivariate logistic regression analyses. The ability of SII to predict outcome was evaluated by the area under the receiver operating characteristic (ROC) curve (AUC).

**Results:**

A total of 1,569 patients with ICH were included, of whom 790 had adverse outcome (50.35%). The Univariate logistic regression analysis showed that SII at any time point within 14 days after admission was significantly associated with adverse outcome. In the multivariate logistic regression analysis, the SII within 7 days after admission was found to be an independent predictor of adverse functional outcome in ICH patients at discharge. The ROC curve demonstrated that compared to other time points, the SII on day 2 after admission exhibited stronger predictive power for the functional outcome of patients with ICH at discharge (AUC:0.733, 95%CI = 0.679–0.787) (sensitivity 47.09%, specificity 87.02%) (OR 1.074, 95%CI = 1.033–1.126, *p* = 0.001).

**Conclusion:**

SII within 7 days after admission, especially on day 2, is independently associated with adverse functional outcome in ICH patients at discharge. This association could be utilized in clinical practice and warrants further investigation.

## Introduction

In low and middle-income countries, stroke is the main reason for mortality and disability and China has the highest rates of stroke worldwide (1, 2). Intracerebral hemorrhage (ICH) makes up 10–15% of all cases of stroke, but contributes to almost 50% of global stroke mortality, and 60–80% of survivors experience serious neurological impairment, which ranks first among all kinds of acute cerebrovascular diseases (3, 4). Due to the aging population, the prevalence of ICH has dramatically increased over the years (5). According to statistics, there are currently at least 3 million new cases of ICH worldwide annually, and this number reached 0.85 million in China in 2019, which imposes a significant burden on the Chinese healthcare system (6–8).

In the area of outcome prediction in ICH, several prognostic tools have been used in clinical practice, including the ICH Score, ICH Grading Scale (ICH-GS), ICH Functional Outcome Score (ICH-FOS), National Institutes of Health Stroke Scale (NIHSS), and Glasgow Coma Scale (GCS) (9–12). However, all these tools may be influenced by clinical decisions, which can impact their predictive value (13). Therefore, no individually score is recommended for predicting ICH prognosis at present (14). In recent years, a number of immune inflammatory makers such as platelet-to-lymphocyte ratio (PLR), neutrophil-to-lymphocyte ratio (NLR) and lymphocyte-to-monocyte ratio (LMR) have shown close associations with functional outcome or mortality after ICH (15–17).

The systemic immune-inflammation index(SII),which integrates platelet, neutrophil and lymphocyte, is a novel immune inflammatory marker that can more comprehensively reflect the balance between immune and inflammatory responses (18).The SII has been proven to have excellent predictive value for many diseases, including myasthenia gravis (19), cardiovascular disease (20), malignant tumor (21), pulmonary embolism (22), acute ischemic stroke (23, 24) and delayed cerebral vasospasm after aneurysmal subarachnoid hemorrhage (25). Recently, some studies have confirmed that the SII is associated with in-hospital mortality, pneumonia, prolonged mechanical ventilation, acute kidney injury, early hematoma expansion, early and long-term functional outcome after ICH, and could be used to independently predict ICH patient prognosis (26–32). To our knowledge, however, most existing research had small sample sizes and only reported one result of the SII on admission. As a consequence, the aim of this study was to investigate the predictive value of SII at different time points for the prognosis of ICH.

## Methods

This was a retrospective case–control study using an ICH database from our medical center for neurological diseases in Deyang city, China. The study protocol was approved by the Institutional Ethics Committee of Deyang People’s Hospital (No. 2023–04-085-K01), and conducted in accordance with the principles outlined in the Declaration of Helsinki. All medical record data were anonymized to protect patient privacy, thereby waiving the requirement for informed consent.

### Patient population

Patients with ICH who were treated at the medical center for neurological diseases in Deyang city between October 2012 and April 2022 were retrospectively enrolled. Patients were included if they met the following criteria: (1) admission diagnosis of ICH according to brain noncontrast computed tomography (NCCT) scan; (2) aged over 18 years; (3) at least one available laboratory test of platelets, neutrophils and lymphocytes after admission. Exclusion criteria were as follows: (1) secondary ICH due to other etiologies such as cerebrovascular malformation, cerebral aneurysm, hemorrhagic cerebral tumor and infarction or coagulopathy; (2) more than 72 h from onset to admission; (3) lack of laboratory test within 72 h after admission; (4) diseases that may affect laboratory test of platelets, neutrophils and lymphocytes on admission, including hemopathy (aplastic anemia, agranulocytosis, or hematological malignancies), infectious or immune diseases; (5) pre-stroke modified Rankin Scale (mRS) score greater than 1 due to any cause; (6) extreme blood cell counts; (7) incomplete data; (8) pregnant women.

### Data collection

A number of data were extracted from the ICH database, including demographic characteristics, clinical features, laboratory tests and imaging data. The baseline variables included age, gender, medical history (hypertension, diabetes, chronic obstructive pulmonary disease, live cirrhosis, heart disease, chronic kidney disease, malignant tumor, history of stroke) and clinical records.

All of the enrolled patients underwent a brain NCCT scan on admission. The volume and position of hemorrhage were calculated based on the first brain NCCT scan. Hematoma volume was measured by the ABC/2 method (5). All laboratory test samples were obtained from patients after admission, and blood parameters were immediately tested by an automatic hematology analyzer (XE-2100, Sysmex Company, Japan), containing platelet count (PLT), absolute neutrophil count (ANC), absolute lymphocyte count (ALC). The SII was computed using the following formula: SII=PLT × ANC/ALC as previously defined (33). The first day of hospitalization was defined as the admission day, the second day as day 1 after admission, and so on.

Functional outcome was assessed using the mRS score, which was evaluated through in-person interviews conducted by an experienced neurosurgeon at hospital discharge. A favorable outcome was defined as an mRS score of 0–3, while an adverse outcome was defined an mRS score of 4–6, which generally represents profound disability or death after ICH, as reported in previous studies (18, 30–32, 34).

### Statistical analysis

Data obtained from multiple laboratory tests within the same period were averaged. All data were double-checked to avoid mistakes before the analysis. The JMP Pro software (version 16.0.0; SAS Institute Inc., Cary, NC, United States) and GraphPad Prism (version 10.1.2; GraphPad Software, San Diego, California, United States) were used for statistical analyses. Based on the results of the normality test, continuous variables were expressed as mean ± standard deviation (Mean ± SD) or medians (interquartile range, IQR). Categorical variables were presented as frequencies (percentages). Differences in demographic characteristics, clinical features, laboratory tests and imaging data among patients with different prognoses were compared by independent student’s t-test, Wilcoxon rank-sum test or χ^2^ test accordingly. Univariate logistic regression analysis was used to confirm the effect of each factor on functional outcome, and variables with *p* < 0.05 were subsequently entered into the multivariate logistic regression model to determine independent risk factors. The predictive value of SII at different time points for adverse outcome was estimated by generating receiver operating characteristic (ROC) curves and calculating the area under the curve (AUC). The optimal cutoff point was determined by the Youden’s Index (sensivitity+specificity-1). In all models mentioned above, SII values were divided by 100 to enhance the readability of the odds ratio (OR). A two-tailed *p* < 0.05 was considered statistically significant.

## Results

### Baseline characteristics

**Figure 1:**
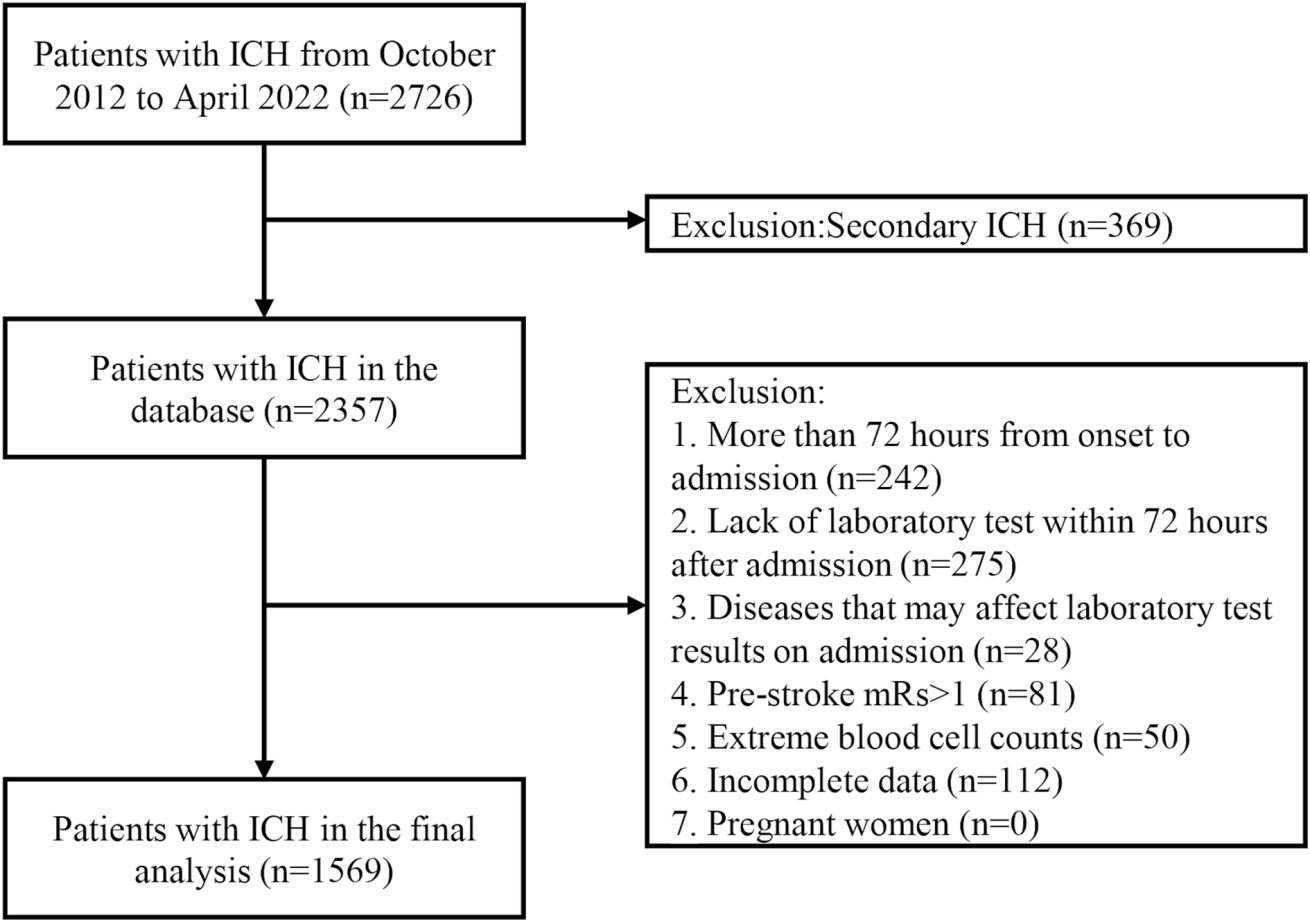
The flowchart of this study. ICH, intracerebral hemorrhage; mRS, modified Rankin Scale.

In total, 2,726 patients with ICH were treated at our medical center for neurological diseases, and 2,357 patients have been consecutively included in the ICH database between October 2012 and April 2022.In this study, we excluded those who had a time interval of more than 72 h from onset to admission (*n* = 242), did not undergo any laboratory tests within 72 h after admission (*n* = 275), had diseases(hematologic, infectious or immune diseases) that may affect laboratory test results on admission (*n* = 28), had pre-stroke mRS score greater than 1 (*n* = 81), extreme blood cell counts (*n* = 50), and incomplete data (*n* = 112). Finally, 1,569 patients were enrolled in the final analysis (Figure 1).

The baseline characteristics of the enrolled patients are presented in Table 1. The median age was 64 (IQR 55–74) years, and 63.67% were male. Among the patients, 1,399(89.17%) had hypertension, 632 (40.28%) had nosocomial pulmonary infection, and 316 (20.14%) underwent surgical treatment after admission. The most common site of hemorrhage was deep (64.95%), including the thalamus and basal ganglia, followed by infratentorial (15.55%), lobar (13.45%), and ventricular (6.05%) locations. The median ICH volume was 13.5 (IQR 5–35.2) ml. Moreover, the SII value fluctuated between 788 × 10^9^/L and 1,113 × 10^9^/L, reaching its highest level of 1113.81 × 10^9^/L (IQR 620.25–1988.01 × 10^9^/L) on the first day after admission.

**Table 1:** Baseline characteristics of patients.

Variables	Total (*n* = 1,569)	Favorable outcome (*n* = 779)	Adverse outcome (*n* = 790)	*p-* value
Age (years)	64 (55–74)	62 (53–71)	67 (57–75)	**<0.001**
Sex (%)				**0.021**
Male	999 (63.67)	518 (66.50)	481 (60.89)	
Female	570 (36.33)	261 (33.50)	309 (39.11)	
Smoking (%)	461 (29.38)	219 (28.11)	242 (30.63)	0.273
Drinking (%)	482 (30.72)	234 (30.04)	248 (31.39)	0.561
Hypertension (%)	1,399 (89.17)	688 (88.32)	711 (90.00)	0.284
Diabetes mellitus (%)	210 (13.38)	95 (12.20)	115 (14.56)	0.170
COPD (%)	48 (3.06)	26 (3.34)	22 (2.78)	0.525
Liver cirrhosis (%)	28 (1.78)	12 (1.54)	16 (2.03)	0.468
Cancer (%)	22 (1.40)	8 (1.03)	14 (1.77)	0.209
Heart disease (%)	75 (4.78)	33 (4.24)	42 (5.32)	0.316
Renal insufficiency (%)	69 (4.40)	17 (2.18)	52 (6.58)	**<0.001**
Prior stroke (%)	112 (7.14)	59 (7.57)	53 (6.71)	0.506
NP (%)	632 (40.28)	230 (29.53)	402 (50.89)	**<0.001**
Surgery (%)	316 (20.14)	98 (12.58)	218 (27.59)	**<0.001**
Admission SBP (mmHg)	173 (154–190)	170 (151–188)	178 (157–191)	**<0.001**
Admission DBP (mmHg)	97 (85–109)	96 (84–107)	98 (86–110)	**0.029**
Admission GCS	10 (6–14)	13 (11–15)	7 (5–10)	**<0.001**
Admission mRS	4 (3–5)	3 (3–4)	5 (4–5)	**<0.001**
Hematoma location (%)				**<0.001**
Deep	1,019 (64.95)	511 (65.60)	508 (64.30)	
Ventricle	95 (6.05)	16 (2.05)	79 (10.00)	
Infratentorial	244 (15.55)	122 (15.66)	122 (15.44)	
Lobar	211 (13.45)	130 (16.69)	81 (10.25)	
Hematoma volume (ml)	13.50 (5.00–35.20)	6.80 (2.70–15.70)	26.55 (11.18–50.00)	**<0.001**
SII (×10^9^/L)				
Admission day	1009.97 (508.87–1863.90)	837.69 (465.49–1520.74)	1242.05 (560.96–2132.39)	**<0.001**
Day 1	1113.81 (620.25–1988.01)	745.16 (465.52–1250.22)	1384.83 (835.19–2499.88)	**<0.001**
Day 2	1003.24 (604.59–1693.49)	695.25 (421.52–1173.26)	1337.33 (749.69–2150.18)	**<0.001**
Day 3-4	924.38 (505.85–1651.43)	701.06 (421.71–1243.95)	1224.96 (673.66–2263.42)	**<0.001**
Day 5-7	788.57 (465.00–1302.77)	629.96 (407.46–1022.59)	1034.95 (669.71–1669.36)	**<0.001**
Day 8-10	863.09 (507.02–1234.34)	740.79 (446.07–1164.01)	1087.15 (694.73–1680.00)	**<0.001**
Day 11-14	788.90 (425.50–1502.21)	629.92 (369.98–1208.78)	1583.61 (1222.32–3098.39)	**<0.001**

COPD, chronic obstructive pulmonary disease; NP, nosocomial pneumonia; SBP, systolic blood pressure; DBP, diastolic blood pressure; GCS, Glasgow Coma Scale; mRS, modified Rankin Scale; SII, systemic immune-inflammation index. The significance of bold data: *p* < 0.05.

### Factors associated with adverse outcome

At discharge, 790 (50.35%) patients achieved adverse outcome. Compared with the favorable outcome group, the adverse outcome group included more females, older patients, those with renal insufficiencies, nosocomial pulmonary infections, intraventricular hemorrhages, surgeries, larger ICH volumes, higher systolic and diastolic blood pressures, lower GCS and higher mRS scores on admission (all *p* < 0.05), as shown in Table 1. The SII was significantly higher in the adverse outcome group than in the favorable outcome group at all time points (all *p* < 0.001; see Table 1).

### Logistic regression analysis of factors related to adverse outcome

Univariate logistic regression analysis showed that increasing age, female, renal insufficiency, nosocomial pulmonary infection, surgery, intraventricular hemorrhage, large ICH volume, high systolic and diastolic blood pressure, low GCS and high mRS scores on admission, especially SII at any time point within 14 days after admission were significantly associated with adverse outcome (all *p* < 0.05; see Table 2).

**Table 2:** Univariate logistic regression analysis of factors related to adverse outcome.

Predictors	OR (95% CI)	*p-* value
Age	1.023 (1.015–1.032)	**<0.001**
Sex (female vs. male)	1.275 (1.038–1.568)	**0.021**
Smoking	1.129 (0.909–1.404)	0.273
Drinking	1.066 (0.860–1.321)	0.561
Hypertension	1.190 (0.866–1.640)	0.284
Diabetes mellitus	1.227 (0.917–1.645)	0.170
COPD	0.830 (0.462–1.476)	0.526
Liver cirrhosis	1.321 (0.624–2.874)	0.470
Cancer	1.739 (0.740–4.375)	0.215
Heart disease	1.269 (0.797–2.037)	0.317
Renal insufficiency	3.158 (1.848–5.670)	**<0.001**
Prior stroke	0.878 (0.596–1.289)	0.506
NP	2.473 (2.011–3.047)	**<0.001**
Surgery	2.648 (2.042–3.456)	**<0.001**
Admission SBP	1.009 (1.005–1.012)	**<0.001**
Admission DBP	1.007 (1.001–1.012)	**0.018**
Admission GCS	0.641 (0.615–0.667)	**<0.001**
Admission mRS	9.540 (7.787–11.820)	**<0.001**
Hematoma location (vs. lobar)		
Deep	1.596 (1.181–2.167)	**0.003**
Ventricle	7.924 (4.429–14.937)	**<0.001**
Infratentorial	1.605 (1.106–2.338)	**0.013**
Hematoma volume	1.050 (1.043–1.057)	**<0.001**
SII (per 100 units)		
Admission day	1.029 (1.020–1.039)	**<0.001**
Day 1	1.071 (1.045–1.100)	**<0.001**
Day 2	1.118 (1.079–1.164)	**<0.001**
Day 3-4	1.077 (1.051–1.106)	**<0.001**
Day 5-7	1.096 (1.065–1.131)	**<0.001**
Day 8-10	1.075 (1.033–1.124)	**<0.001**
Day 11-14	1.038 (1.012–1.069)	**0.008**

OR, odds ratio; CI, confidence interval; COPD, chronic obstructive pulmonary disease; NP, nosocomial pneumonia; SBP, systolic blood pressure; DBP, diastolic blood pressure; GCS, Glasgow Coma Scale; mRS, modified Rankin Scale; SII, systemic immune-inflammation index. The significance of bold data: *p* < 0.05.

**Figure 2:**
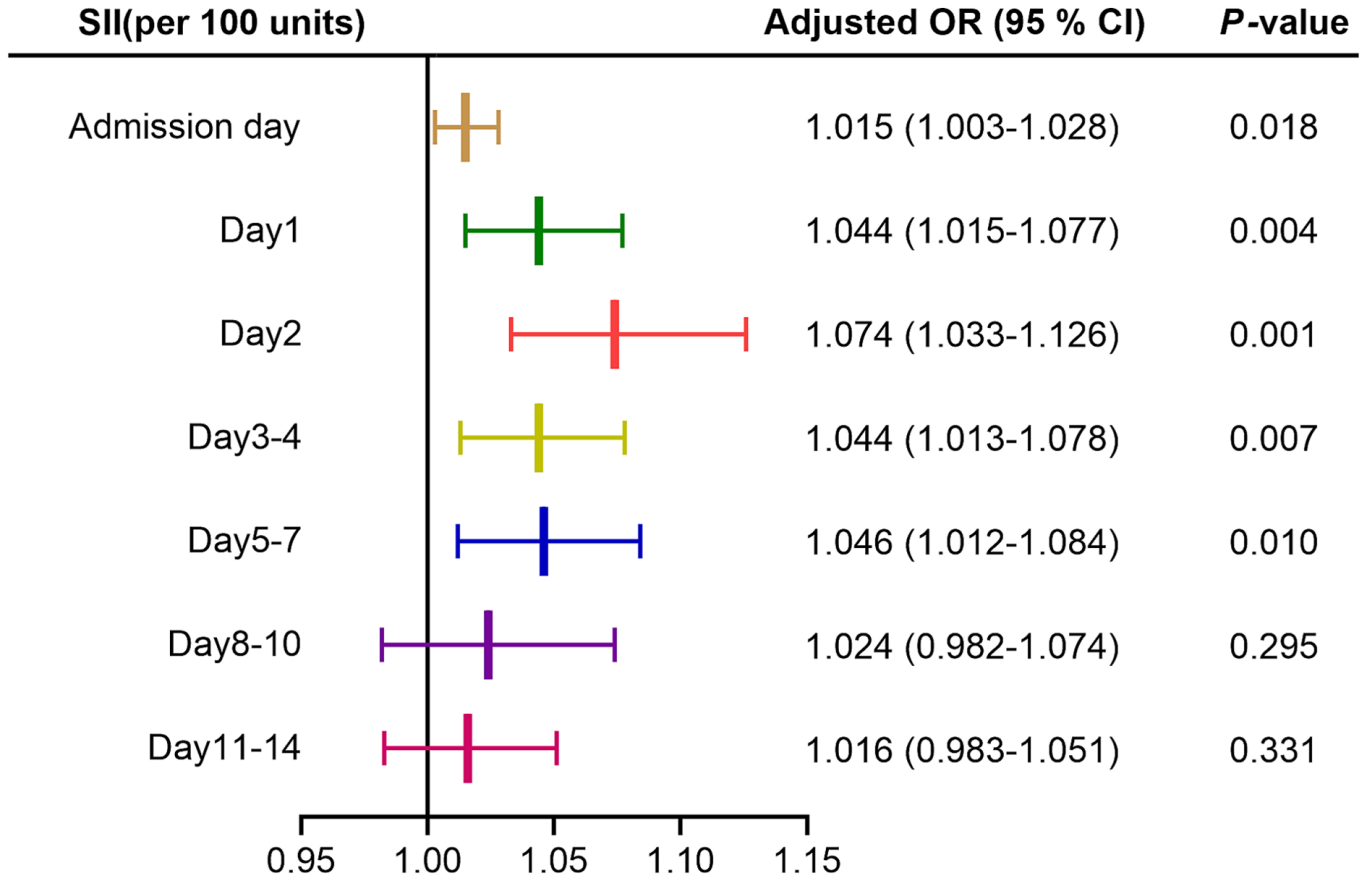
Forest map of multivariate logistic regression analysis for SII. Adjusted for age, gender, nosocomial pulmonary infection, surgery, systolic and diastolic blood pressure, GCS and mRS score on admission, hematoma location and volume, renal insufficiency. SII, systemic immune-inflammation index; OR, odds ratio; CI, confidence interval.

In multivariate logistic regression analysis, after adjusting for age, gender, nosocomial pulmonary infection, surgery, systolic and diastolic blood pressure, GCS and mRS score on admission, hematoma location and volume, renal insufficiency; SII on admission day, day 1, day 2, day 3–4, day 5–7 after admission were able to independently predict the functional outcome of ICH patients at discharge, while day 8–10 and 11–14 after admission did not, as shown in Figure 2. In addition, hematoma volume was an independent predictor of the functional outcome of ICH patients at discharge in all models (all *p* < 0.05; see Supplementary Table 1).

### Receiver operating characteristic curves analysis for predicting adverse outcome by SII

**Figure 3:**
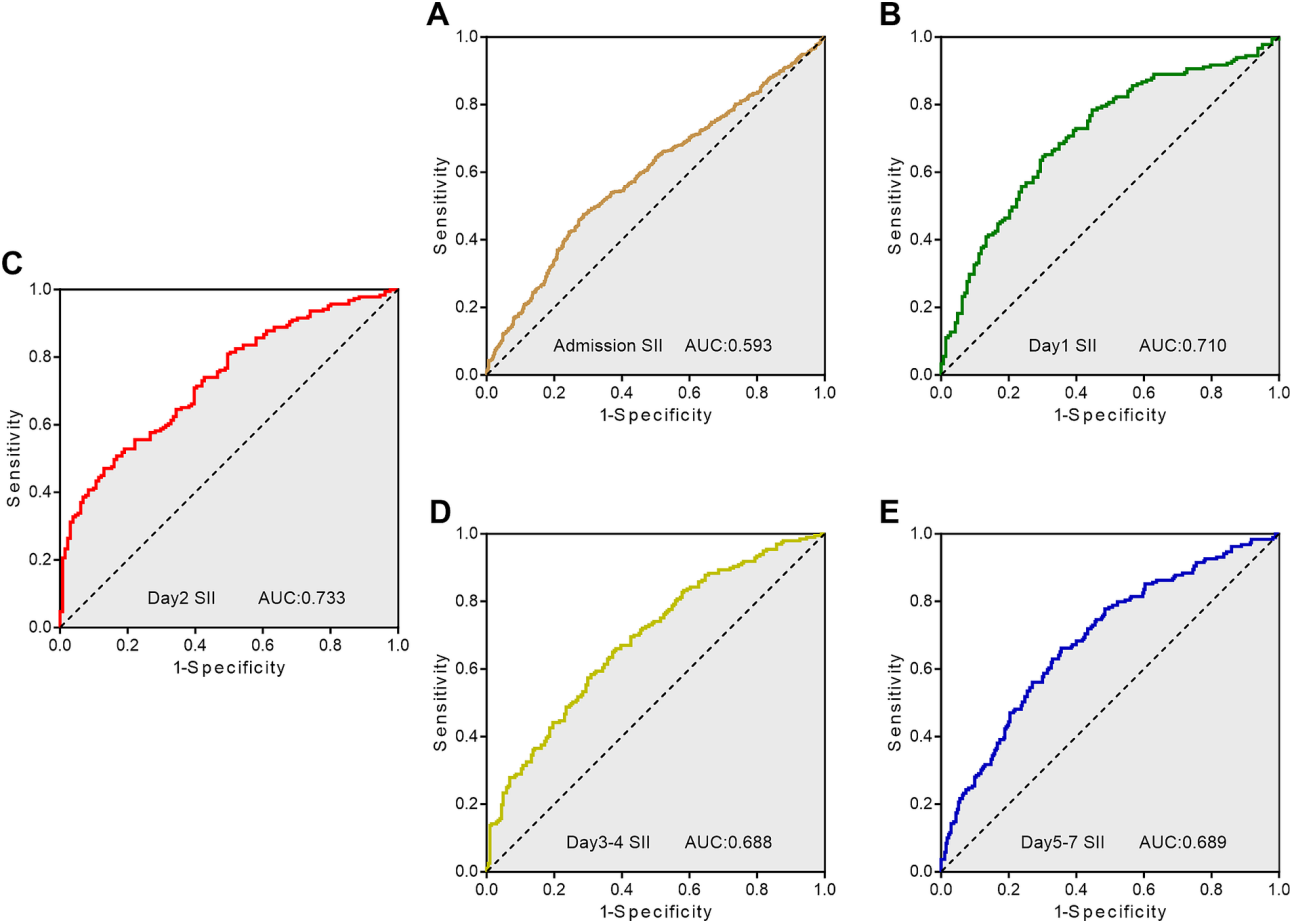
Receiver operating characteristic curves showing the predictive value of SII at different time points for adverse outcome. **(A)** ROC curve for SII on admission day; **(B)** ROC curve for SII on day 1; **(C)** ROC curve for SII on day 2; **(D)** ROC curve for SII on day 3–4; **(E)** ROC curve for SII on day 5–7. SII, systemic immune-inflammation index; AUC, area under the curve; ROC, Receiver operating characteristic.

According to the ROC curves analysis, the AUC values of SII gradually increased with an increase in the length of hospital stay, reaching their highest point on day 2 after admission, and then declining (Figure 3). Compared to other time points, the SII on day 2 after admission showed stronger predictive power for the functional outcome of ICH patients at discharge (AUC 0.733, 95%CI = 0.679–0.787) (sensitivity 47.09%, specificity 87.02%), and the optimal cutoff point was 1,430 × 10^9^/L, as shown in Table 3.

**Table 3:** Predictive power of SII related to adverse outcome.

SII (per 100 units)	AUC (95% CI)	*p-* value	Cutoff value	Sensitivity	Specificity
Admission day	0.593 (0.563–0.624)	<0.001	13.424	0.475	0.713
Day 1	0.710 (0.654–0.767)	<0.001	11.279	0.646	0.699
Day 2	0.733 (0.679–0.787)	<0.001	14.301	0.471	0.870
Day 3-4	0.688 (0.637–0.740)	<0.001	9.010	0.660	0.618
Day 5-7	0.689 (0.640–0.738)	<0.001	8.183	0.661	0.644

SII, systemic immune-inflammation index; AUC, area under the curve; CI, confidence interval.

## Discussion

Currently, the majority of studies on SII in ICH patients have been based on a single blood detection without dynamic testing after admission (26–28, 30, 32, 35). In this study, we utilized the hospital’s scientific research big data platform to retrieve and analyze the data of patients diagnosed with ICH over the past decade. We found that SII within 14 days after admission was significantly associated with adverse outcome at discharge. Additionally, SII within the first 7 days after admission could independently predict the functional outcome of ICH patients at discharge in multivariate logistic regression analysis. Furthermore, as the number of hospitalization days increased, the predictive power of SII gradually enhanced and reached its highest point on day 2 after admission with an optimal cutoff point of 1,430 × 10^9^/L, and then began to decline. These findings suggest that SII could be an important new indicator for prognostic prediction, risk stratification and assessment of disease severity in ICH patients (18). To our knowledge, this was the first study to report on the dynamic changes of SII in ICH patients during the 14 days after admission and assess the predictive value of SII for functional outcome at discharge following ICH.

The brain damage after ICH is generally classified into two phases: primary and secondary damage. The primary brain damage results from mechanical injury to brain tissue caused by the hematoma, the severity of which mainly depends on the size, location and extent of the hematoma (36). As reported by previous research, hematoma volume could independently predict the prognosis of ICH patients (18, 31, 32), which was also verified in our study.

The extravasation of blood components into the brain tissue leads to secondary brain damage, while the neuroinflammatory response mediated by immune cells plays a pivotal role in the initiation and progression of this process (18, 37, 38). After 1 to 1.5 h following ICH, microglia, the innate immune cells in brain tissue, are activated by blood components exuded from the hematoma (39). Meanwhile, macrophages also migrate from the bloodstream into the central nervous system (40). Activated microglia/macrophages release a variety of pro-inflammatory factors, chemokines, reactive oxygen species (ROS) and matrix metalloproteinases (MMPs), thereby facilitating the infiltration of peripheral inflammatory cells, disrupting blood–brain barrier integrity, inducing neuronal apoptosis, and ultimately leading to brain injury and neurological dysfunction (41). Neutrophils as the earliest leukocytes infiltrating into the brain from peripheral blood (42), exhibit a gradual increase within a few hours after stroke onset and maintain an elevated level for a week (43). Neutrophils exacerbate brain injury by secreting tumor necrosis factor *α* (TNF-α), ROS, and matrix metalloproteinase 9(MMP-9) (44). In a previous pathological investigation of brain tissue from ICH patients, perihematomal neutrophils were observed on the first day, while pronounced neutrophil infiltration was noted from the second to twelfth day (45). Clinical studies have demonstrated that an elevated neutrophil count on admission is independently associated with a worse prognosis after ICH (46, 47).

An excessive and persistent inflammatory response following stroke may lead to immune system exhaustion, resulting in systemic immunosuppression, which is commonly referred to as stroke-induced immunodepression syndrome (SIDS) (27, 48). The primary clinical presentation of SIDS is characterized by rapid and sustained suppression of cellular immunity, accompanied by inactivation and reduction of lymphocytes (49). Immunodepression promptly initiates within 24 h following the onset of stroke and persists for several weeks (49). This phenomenon may potentially compromise the body’s ability to resist pathogenic bacteria, increase susceptibility to infections, and contribute to an unfavorable prognosis (48). The observational study has shown that low admission absolute lymphocyte count is an independent risk factor for unfavorable outcome at 3 months in ICH patients (50). In addition, platelets undergo activation during the hemostasis process following ICH, leading to an elevation in platelet count and inducing a hypercoagulable state (16). Activated platelets can cause a severe inflammatory response by inducing the release of inflammatory cytokines (51). Consequently, high platelet count levels may indicate an exacerbation of the inflammatory response (30), which is closely associated with an unfavorable prognosis.

In conclusion, neutrophils, platelets and lymphocytes are strongly associated with the neuroinflammatory response after ICH. Their respective counts undergo dynamic changes throughout this process. Considering the susceptibility of individual blood cell parameters to factors such as race, excessive dehydration and specimen handling (18), the utilization of a comprehensive inflammation index, like SII, may provide a more precise reflection of the intricacy of the inflammatory response (32). Studies have shown that SII provides more accurate prognostic predictions for patients with ICH (18, 32). Furthermore, the SII index is derived from indicators obtained through blood routine examination, which is an essential hematological examination for almost all ICH patients. Its convenience and cost-effectiveness contribute to alleviating medical burden and facilitating its widespread adoption.

According to previous research, laboratory tests may not accurately reflect the inflammatory immune response in the initial hours following onset (52), which could compromise the predictive value of a single SII test on admission. In light of this, several scholars have proposed that greater emphasis should be placed on the more precise examination of inflammatory immune markers, such as SII, in stroke patients (31). Therefore, we investigated the dynamic changes of SII and found that SII on day 1 after admission, as well as day 5–7, independently predicted the functional outcome of ICH patients at discharge with superior predictive capacity compared to admission day, which is consistent with the existing literature (18, 31). Notably, our study revealed a novel finding that SII on day 2 after admission exhibited greater predictive capacity compared to other time points during the first 7 days post-admission. This finding could provide valuable guidance for the clinical application of SII. Furthermore, our study has the largest sample size to date and encompasses nearly all types of ICH patients compared to previous investigations. These aspects are strengths of our study.

There are several limitations in our study. First, this study was a retrospective analysis conducted at a single center, which may introduce potential biases. Second, not all patients were hospitalized for more than 14 days or underwent multiple blood cell tests. The other time groups had a greater number of time points compared to the initial 2 days after admission, while the sample size on day 11–14 was limited. All these factors might have potentially influenced our findings. Third, our study solely focused on short-term functional outcome at discharge without evaluating long-term functional outcome. Moreover, the cutoff point may vary depending on factors such as population, geographical region, or device. Therefore, further multicenter prospective studies with large sample sizes may be required to verify our findings.

## Conclusion

Our findings suggest that SII within 7 days after admission, especially on day 2, is significantly associated with poor functional outcome at discharge in patients with ICH, and it may be a reliable predictor for prognosis. Due to its convenience and cost-effectiveness, SII can be easily integrated into routine clinical practice.

## Data Availability

The original contributions presented in the study are included in the article/Supplementary material, further inquiries can be directed to the corresponding author.
