# Supplementary material for: Predictive value of the dynamic systemic immune-inflammation index in the prognosis of patients with intracerebral hemorrhage: a 10-year retrospective analysis
Source: Front Neurol. 2024 Oct 8;15:1444744. doi: 10.3389/fneur.2024.1444744 (PMC11497262; doi:10.3389/fneur.2024.1444744)
Supplement: Supplementary file 1 [file Table_1.DOCX]

| **Predictors** | | | | **OR (95% CI)** | | | ***P-*value** | **OR (95% CI)** | | ***P-*value** | | **OR (95% CI)** | | ***P-*value** |
| --- | --- | --- | --- | --- | --- | --- | --- | --- | --- | --- | --- | --- | --- | --- |
| Age | | | | 1.034(1.019-1.049) | | | **<0.001** | 1.030(1.003-1.060) | | **0.033** | | 1.052(1.021-1.086) | | **0.001** |
| Sex (female vs. male) | | | | 1.306(0.932-1.831) | | | 0.121 | 1.594(0.858-2.995) | | 0.143 | | 1.551(0.766-3.196) | | 0.226 |
| NP | | | | 1.724(1.234-2.413) | | | **0.001** | 2.354(1.274-4.405) | | **0.007** | | 1.464(0.713-2.977) | | 0.294 |
| Surgery | | | | 0.295(0.180-0.479) | | | **<0.001** | 0.427(0.180-0.979) | | **0.047** | | 0.500(0.190-1.280) | | 0.152 |
| Admission SBP | | | | 1.003(0.995-1.011) | | | 0.456 | 1.000(0.987-1.013) | | 0.997 | | 0.994(0.979-1.009) | | 0.406 |
| Admission DBP | | | | 1.001(0.988-1.014) | | | 0.871 | 1.011(0.988-1.035) | | 0.332 | | 1.018(0.995-1.042) | | 0.130 |
| Admission GCS | | | | 0.862(0.794-0.935) | | | **<0.001** | 0.914(0.786-1.061) | | 0.239 | | 0.915(0.764-1.093) | | 0.327 |
| Admission mRS | | | | 3.751(2.507-5.669) | | | **<0.001** | 3.236(1.534-7.037) | | **0.002** | | 4.769(2.014-12.095) | | **<0.001** |
| Hematoma location (vs. lobar) | | | |  | | | | | | | | | | |
| Deep | | | | 3.174(1.897-5.404) | | | **<0.001** | 5.258(2.132-13.847) | | **<0.001** | | 5.864(2.092-17.608) | | **0.001** |
| Ventricle | | | | 16.510(6.446-44.244) | | | **<0.001** | 12.939(2.059-106.950) | | **0.010** | | 18.146(3.112-127.367) | | **0.002** |
| Infratentorial | | | | 4.875(2.514-9.588) | | | **<0.001** | 5.375(1.451-20.709) | | **0.013** | | 3.786(0.882-16.769) | | 0.075 |
| Renal insufficiency | | | | 6.337(2.792-14.960) | | | **<0.001** | 6.990(1.385-56.508) | | **0.034** | | 10.546(2.653-52.662) | | **0.002** |
| Hematoma volume | | | | 1.054(1.042-1.067) | | | **<0.001** | 1.033(1.014-1.053) | | **<0.001** | | 1.039(1.017-1.064) | | **<0.001** |
| SII  (per 100 units) | | Admission Day | | 1.015(1.003-1.028) | | | **0.018** |  | | | | | | |
|  |  | Day1 | |  | | | | 1.044(1.015-1.077) | | **0.004** | |  | | |
|  |  | Day2 | |  | | | | | | | | 1.074(1.033-1.126) | | **0.001** |
| **Predictors** | | | **OR (95% CI)** | | ***P-*value** | **OR (95% CI)** | | ***P-*value** | **OR (95% CI)** | | ***P-*value** | | **OR (95% CI)** | ***P-*value** |
| Age | | | 1.035(1.011-1.061) | | **0.005** | 1.045(1.020-1.071) | | **<0.001** | 1.020(0.985-1.057) | | 0.263 | | 1.062(0.999-1.135) | 0.059 |
| Sex (female vs. male) | | | 1.347(0.749-2.438) | | 0.321 | 0.672(0.382-1.172) | | 0.164 | 2.722(1.257-6.067) | | **0.012** | | 0.350(0.066-1.565) | 0.186 |
| NP | | | 1.858(1.061-3.260) | | **0.030** | 2.249(1.326-3.839) | | **0.003** | 1.501(0.680-3.297) | | 0.311 | | 1.426(0.380-5.277) | 0.591 |
| Surgery | | | 0.767(0.353-1.652) | | 0.499 | 0.511(0.232-1.105) | | 0.091 | 0.544(0.134-2.157) | | 0.386 | | 0.199(0.013-2.341) | 0.217 |
| Admission SBP | | | 1.001(0.988-1.014) | | 0.896 | 0.993(0.980-1.006) | | 0.301 | 0.995(0.977-1.012) | | 0.534 | | 1.007(0.977-1.039) | 0.653 |
| Admission DBP | | | 1.002(0.982-1.022) | | 0.882 | 1.015(0.993-1.038) | | 0.185 | 1.008(0.983-1.034) | | 0.547 | | 0.993(0.941-1.046) | 0.803 |
| Admission GCS | | | 0.880(0.763-1.012) | | 0.075 | 0.943(0.822-1.079) | | 0.395 | 0.911(0.745-1.109) | | 0.357 | | 0.868(0.576-1.303) | 0.491 |
| Admission mRS | | | 2.228(1.134-4.425) | | **0.021** | 5.188(2.690-10.426) | | **<0.001** | 3.245(1.276-8.749) | | **0.016** | | 4.332(0.766-28.723) | 0.106 |
| Hematoma location  (vs. lobar) | | |  | | | | | | | | | | | |
| Deep | | | 4.652(1.887-12.433) | | **0.001** | 2.523(1.062-6.360) | | **0.042** | 3.235(0.966-12.631) | | 0.071 | | 2.766(0.457-22.671) | 0.296 |
| Ventricle | | | 20.473(4.709-100.230) | | **<0.001** | 20.844(4.396-110.889) | | **<0.001** | 8.549(1.177-70.933) | | **0.039** | | 10.664(0.106-744.930) | 0.266 |
| Infratentorial**^#^** | | | 11.022(3.127-41.502) | | **<0.001** | 1.774(0.455-6.834) | | 0.405 | 2.251(0.310-15.609) | | 0.410 | | - | - |
| Renal insufficiency | | | 6.108(1.684-23.890) | | **0.007** | 7.751(2.130-31.207) | | **0.003** | 13.464(1.918-108.421) | | **0.010** | | 2.962(0.027-199.936) | 0.645 |
| Hematoma volume | | | 1.050(1.031-1.071) | | **<0.001** | 1.047(1.026-1.070) | | **<0.001** | 1.054(1.021-1.090) | | **0.001** | | 1.065(1.004-1.141) | **0.048** |
| SII  (per 100 units) | Day3-4 | | 1.044(1.013-1.078) | | **0.007** |  | | | | | | | | |
|  | Day5-7 | |  | | | 1.046(1.012-1.084) | | **0.010** |  | | | | | |
|  | Day8-10 | |  | | | | | | 1.024(0.982-1.074) | | 0.295 | |  | |
|  | Day11-14 | |  | | | | | | | | | | 1.016(0.983-1.051) | 0.331 |

**Supplementary Table 1** **Multivariate logistic regression analysis of factors related to adverse outcome.**

**^#^** Infratentorial ICH could not be compared with other sites due to limited sample size on day 11-14.

OR, odds ratio; CI, confidence interval; NP, nosocomial pneumonia; SBP, systolic blood pressure; DBP, diastolic blood pressure; GCS, Glasgow Coma Scale; mRS, modified Rankin Scale; SII, systemic immune-inflammation index. The significance of bold data: P < 0.05.
